# Red Cell Distribution Width at Diagnosis Reflects Advanced Disease While Dynamic Changes Predict Survival at Relapse in Multiple Myeloma: A Retrospective Study

**DOI:** 10.7759/cureus.75662

**Published:** 2024-12-13

**Authors:** Elif Yigit Ayhan, Ibrahim E Pinar, Vildan Ozkocaman, Fahir Ozkalemkas

**Affiliations:** 1 Department of Internal Medicine, Bursa Uludag University, Bursa, TUR; 2 Department of Internal Medicine, Isparta City Hospital, Isparta, TUR

**Keywords:** autologous stem cell transplantation, multiple myeloma, prognosis, red cell distribution width, survival

## Abstract

Introduction

Multiple myeloma (MM) is a complex plasma cell malignancy characterized by clonal proliferation and monoclonal immunoglobulin production. Despite the availability of several prognostic markers for MM, many are challenging to implement routine clinical practice due to cost, complexity, or lack of standardization. Red cell distribution width (RDW), a cost-effective and routinely measured parameter in complete blood counts, has gained increasing attention as a prognostic marker due to its association with disease severity and outcomes in MM. This study investigates the prognostic utility of RDW in MM, focusing on its relationship with patient outcomes, particularly in those undergoing autologous stem cell transplantation (ASCT).

Methods

This retrospective study included 218 patients diagnosed with MM between 2010 and 2018. Demographic, clinical, and laboratory data, including RDW levels at ASCT and first relapse, were collected. Patients were stratified into high (>16.5%) and low (≤16.5%) RDW groups. The impact of RDW levels and their changes on progression-free survival (PFS) and overall survival (OS) were analyzed using Kaplan-Meier and log-rank tests.

Results

Higher RDW levels at diagnosis were significantly associated with advanced disease stages, notably R-ISS stage 3 (p=0.022). While no significant survival differences were observed based on baseline RDW levels, dynamic changes in RDW from diagnosis to first relapse were strongly prognostic. Patients maintaining low RDW had the longest PFS (37 months) and OS (88.8 months), whereas those transitioning from low to high RDW experienced the shortest PFS (nine months) and OS (40.6 months). At relapse, patients with low RDW demonstrated superior outcomes (PFS: 34 vs. 14 months, OS: 81.2 vs. 40.6 months; p<0.001).

ASCT markedly improved survival outcomes, with longer PFS (p=0.028) and OS (p<0.001). Higher hemoglobin levels (>10 g/dL) were also associated with extended PFS (p=0.038). Reassessing RDW prior to ASCT did not yield significant differences, suggesting that the prognostic value of RDW lies in its dynamic changes, particularly around relapse events.

Conclusions

RDW levels at diagnosis reflect advanced disease stages in MM but are not independent predictors of survival. However, dynamic changes in RDW, particularly from diagnosis to relapse, highlight its potential as a robust marker for monitoring disease progression and relapse risk.

ASCT remains a cornerstone of MM management, significantly improving survival outcomes and complementing RDW trends in prognosis. Standardizing RDW thresholds and integrating its dynamic trends into clinical workflows could enhance risk stratification and personalized treatment strategies. Prospective, multi-center trials are essential to validate these findings and establish RDW's role in comprehensive prognostic frameworks for MM.

## Introduction

Multiple myeloma (MM) is a malignant plasma cell disorder characterized by the uncontrolled proliferation of clonal cells secreting monoclonal immunoglobulins. Although MM accounts for only 10-15% of hematologic malignancies, its heterogeneous nature poses significant challenges in treatment and prognosis [1]. The disease manifests with a wide spectrum of clinical presentations; while many patients exhibit a subacute course, others experience acute symptoms requiring immediate therapeutic intervention. Assessing the patient’s clinical status prior to treatment initiation is essential for setting realistic expectations and designing tailored therapeutic strategies.

Over the years, numerous prognostic markers have been proposed for MM, focusing on cellular and molecular factors influencing disease progression and outcomes [2-4]. However, many of these markers are expensive, technically complex, and lack standardization, limiting their use in routine clinical practice. This underscores the need for accessible, cost-effective tools. Prognostic factors in MM are multifactorial, encompassing patient characteristics, disease stage, and underlying biology [5]. Established indicators include clinical, laboratory, immunophenotypic, and cytogenetic features, which are often integrated into widely used staging systems [6,7].

Amid these challenges, red cell distribution width (RDW), a routinely measured and cost-effective parameter in complete blood counts, has emerged as a potential prognostic tool in MM. Traditionally employed to assess erythrocyte size variability, RDW is now recognized as a marker of ineffective erythropoiesis, often driven by chronic inflammation and neurohumoral activation. Elevated RDW levels have been linked to adverse outcomes in various conditions, including cancer, acute lung diseases, kidney disorders, and cardiovascular diseases, where it has been associated with higher mortality [8-10].

These associations have fueled a growing interest in RDW as a prognostic indicator in MM. While several studies have explored its predictive value, findings remain limited and sometimes inconsistent [2,3]. This study seeks to elucidate the prognostic significance of RDW in MM patients, with a particular emphasis on its predictive value in those undergoing autologous stem cell transplantation (ASCT). By examining RDW as a prognostic tool, we aim to clarify its role in routine practice and its value in guiding treatment decisions for MM patients.

## Materials and methods

This retrospective study included 218 patients aged 18 years or older, diagnosed with MM based on International Myeloma Working Group criteria and treated at the Hematology Department of Bursa Uludag University Faculty of Medicine between January 2010 and December 2018 [11]. Comprehensive data on demographic characteristics, disease-related variables, laboratory results, and pathological findings were systematically collected. Data regarding the application of ASCT and pre-transplant RDW levels were also included in the analysis.

To minimize confounding, patients with conditions known to influence RDW levels, such as iron deficiency anemia, chronic inflammation, nutritional deficiencies (e.g., vitamin B12 and folate), chronic kidney disease, or other hematological disorders, were excluded. In MM cases, anemia was considered an inherent aspect of the disease that was in line with established diagnostic criteria. Patients were stratified into two groups based on RDW levels, determined using our institution’s laboratory reference range: high RDW (>16.5%) and low RDW (≤16.5%). Disease staging was performed using both the International Staging System (ISS) and the Revised International Staging System (R-ISS). The hemoglobin/RDW ratio (HRR) was assessed with a cutoff value of 0.61, as proposed by Baysal et al. [12].

For survival analyses, overall survival (OS) was defined as the time from diagnosis to death or last follow-up, and progression-free survival (PFS) was determined retrospectively using clinical or radiographic evidence of disease progression or relapse. Patients with incomplete data were excluded from the study.

Statistical analysis

The normality of distribution for continuous variables was assessed using the Shapiro-Wilk test. Continuous variables were expressed as mean ± standard deviation (SD) for normally distributed data or as median (minimum-maximum) for non-normally distributed data. Categorical variables were presented as frequencies (n) and percentages (%). Independent samples t-tests were used to compare normally distributed continuous variables, while the Mann-Whitney U test was applied for non-normally distributed data. As appropriate, categorical variables were analyzed using the Chi-square test, Fisher-Freeman-Halton test, or Fisher’s exact test.

Survival analyses for PFS and OS were conducted using the Kaplan-Meier method, and survival curves were compared using the log-rank test. All statistical analyses were performed using SPSS software (IBM SPSS Statistics for Windows, Version 23.0. Armonk, NY: IBM Corp.). Statistical significance was defined as a Type I error rate of 5% (p<0.05).

## Results

The study cohort consisted of 218 patients diagnosed with MM, with a mean age of 61.55 years, of whom 55% were male. Baseline demographic and clinical characteristics, including staging, are presented in Table 1. Most patients (79.8%) received bortezomib-based therapy as their primary treatment regimen, with other therapeutic approaches used less frequently.

**Table 1:** Demographic and baseline characteristics of the patients ISS: international staging system, R-ISS: revised international staging system, LDH: lactate dehydrogenase, SD: standard deviation

Parameters	Values
Age (years, mean ± SD)	61.55±10.69
Gender	
Female	98 (45%)
Male	120 (55%)
Diagnosis	
IgG myeloma	119 (54.6%)
IgA myeloma	43 (19.7%)
Light chain myeloma	52 (23.8%)
Others	4 (1.8%)
ISS	
Stage 1	36 (16.5%)
Stage 2	70 (32.1%)
Stage 3	112 (51.4%)
R-ISS	
Stage 1	28 (12.8%)
Stage 2	162 (74.3%)
Stage 3	28 (12.8%)
Hemoglobin (g/dL, median (range))	10.1 (1.6-17)
Creatinine (mg/dL, median (range))	1 (0.5 -12)
Calcium (mg/dL, median (range))	9.7 (7.1-17.8)
Albumin (g/dL, median (range))	3.3 (1.4-12.5)
LDH (U/L, median (range))	176.5 (37-1712)
Beta-2 microglobulin (ng/mL, median (range))	5.20 (1.50-50)
Sedimentation (mm/hour, median (range))	47 (2-120)

Among the cohort, 38% of patients (n=83) were classified into the high RDW group. While ISS staging did not show significant differences between RDW groups at diagnosis (p=0.207), the R-ISS revealed a significant association, with a higher proportion of stage 3 patients in the high RDW group (p=0.022, Table 2).

**Table 2:** RDW group distribution of MM patients at the time of diagnosis ^a^T-test for independent dual sample ^b^Chi-square test ^c^Fisher-Freeman-Halton test ^d^Fisher’s exact Chi-square test Low RDW (≤16.5%) represents patients with RDW ≤16.5%, while high RDW (>16.5%) represents patients with RDW >16.5%. ISS: international staging system, R-ISS: revised international staging system, LDH: lactate dehydrogenase, ASCT: autologous stem cell transplant, RDW: red cell distribution width, SD: standard deviation; MM: multiple myeloma

Parameter	Low RDW (n=135)	High RDW (n=83)	p-value
Age (years, mean ± SD)	61.02±10.09	62.42±11.61	0.347^a^
Gender			0.930^b^
Female	61 (45.2%)	37 (44.6%)	
Male	74 (54.8%)	46 (55.4%)	
Diagnosis			0.040^c^
IgG myeloma	76 (56.3%)	43 (51.8%)	
IgA myeloma	19 (14.1%)	24 (28.9%)	
Light chain myeloma	37 (27.4%)	15 (18.1%)	
Others	3 (2.2%)	1 (1.2%)	
ISS			0.207^b^
Stage 1	27 (20%)	9 (10.8%)	
Stage 2	42 (31.1%)	28 (33.7%)	
Stage 3	66 (48.9%)	46 (55.4%)	
R-ISS			0.022^b^
Stage 1	22 (16.3%)	6 (7.2%)	
Stage 2	101 (74.8%)	61 (73.5%)	
Stage 3	12 (8.9%)	16 (19.3%)	
Hemoglobin (g/dL, median (range))	10.3 (1.6-17)	9.6 (6-14.8)	0.006^a^
Platelets (/mm³, median (range))	231 (26.8-1460)	220 (34.1-473)	0.442^a^
Creatinine (mg/dL, median (range))	1 (0.5-8)	1 (0.5-12)	0.785^a^
Calcium (mg/dL, median (range))	9.6 (7.1-17.8)	9.9 (7.9-15)	0.240^a^
LDH (U/L, median (range))	172 (68-1712)	190 (37-567)	0.128^a^
Beta-2 microglobulin (ng/mL, median (range))	5.10 (1.80-50)	5.70 (1.50-50)	0.364^a^
Sedimentation (mm/hour, median (range))	47 (2-120)	46 (2-120)	0.398^a^
ASCT performed	68 (50.4%)	27 (32.5%)	0.010^b^

**Figure 1:**
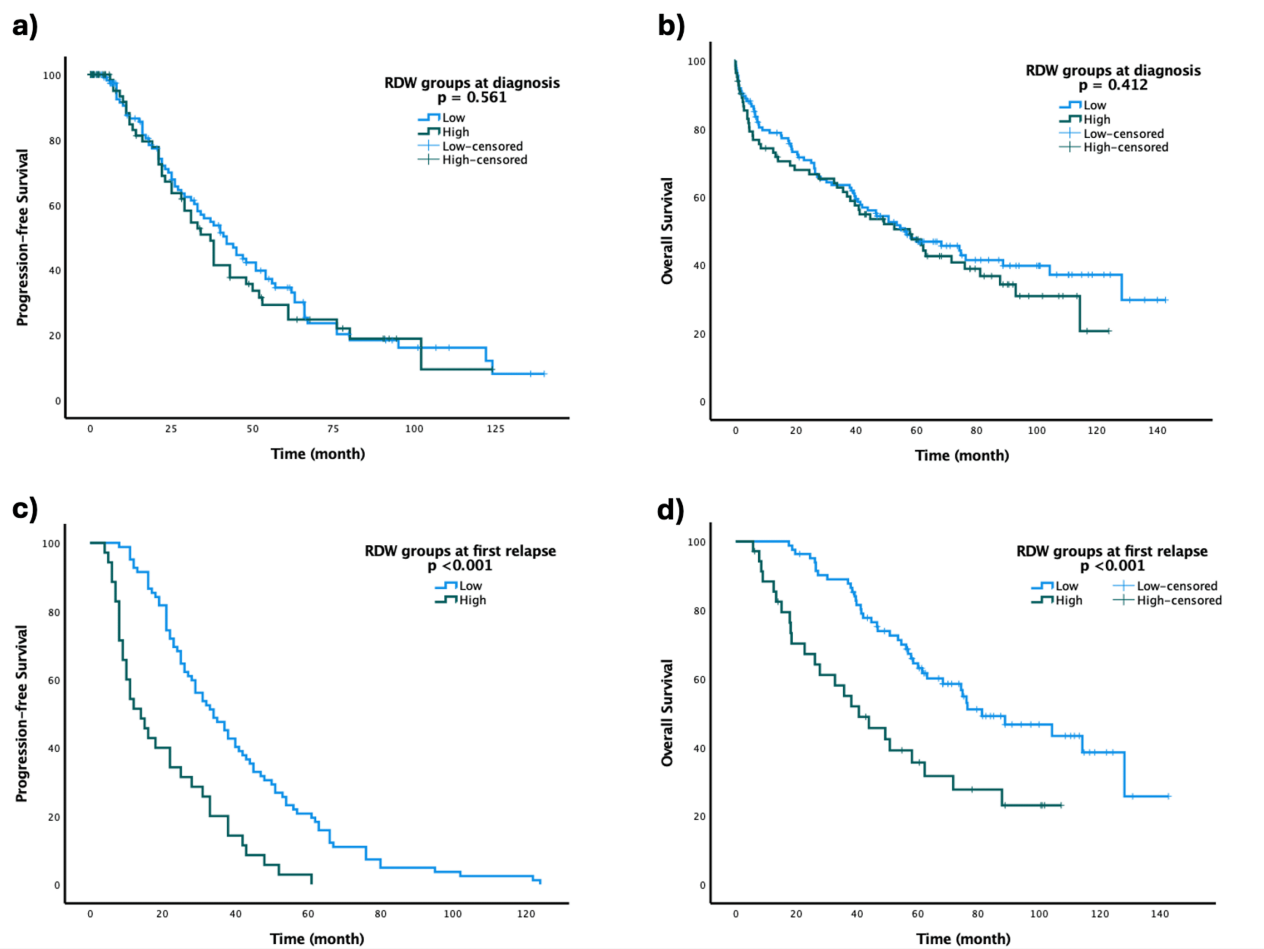
(a) PFS curves by RDW groups at diagnosis, (b) OS curves by RDW groups at diagnosis, (c) PFS curves by RDW groups at first relapse, and (d) OS curves by RDW groups at first relapse Low RDW (≤16.5%) represents patients with RDW ≤16.5%, while high RDW (>16.5%) represents patients with RDW >16.5%. RDW: red cell distribution width; PFS: progression-free survival; OS: overall survival

Patients with light chain myeloma had significantly longer PFS compared to those with IgG or IgA myeloma (p=0.014). Patients who underwent ASCT had a significantly longer PFS (median 45 vs. 31 months, p=0.028), and those with hemoglobin levels >10 g/dL demonstrated substantially better PFS compared to patients with lower levels (p=0.038). However, no significant differences in PFS were observed between high RDW (>16.5%) and low RDW (≤16.5%) groups at diagnosis (p=0.561) (Table 3, Figure 1a).

**Table 3:** PFS outcomes of patients ^x^n=given as the number and ratio among 218 people ^y^Given as the number and ratio within the number of patients at risk ^e^Log-rank test Low RDW (≤16.5%) represents patients with RDW ≤16.5%, while high RDW (>16.5%) represents patients with RDW >16.5%. PFS times are presented as median ± standard error. ISS: international staging system, R-ISS: revised international staging system, ASCT: autologous stem cell transplantation, RDW: red cell distribution width; PFS: progression-free survival; HRR: hemoglobin/RDW ratio

Parameter	Patients at risk, n(%)^x^	Relapses, n(%)^y^	PFS (months)	p-value^e^
Age (years)				0.983
≤60	96 (44)	49 (51)	37±5.96	
>60	122 (56)	74 (60.7)	41±3.39	
Diagnosis				0.014
IgG myeloma	119 (54.6)	65 (54.6)	37±4.27	
IgA myeloma	43 (19.7)	32 (74.4)	34±4.43	
Light chain myeloma	52 (23.9)	18 (34.6)	63±7.55	
Others	4 (1.8)	3 (75)	26±19.93	
Hemoglobin (g/dL)				0.038
Low (≤10)	105 (48.2)	59 (56.2)	31±2.23	
High (>10)	113 (51.8)	59 (52.2)	48±4.53	
RDW groups				0.561
Low	135 (61.9)	74 (54.8)	42±4.57	
High	83 (38.1)	44 (53)	37±3.59	
ISS				0.607
Stage 1	36 (16.5)	26 (72.2)	37±7.79	
Stage 2	70 (32.1)	41 (58.6)	40±1.87	
Stage 3	112 (51.4)	51 (45.5)	33±8.24	
R-ISS				0.217
Stage 1	28 (12.8)	20 (71.4)	37±9.96	
Stage 2	162 (74.3)	85 (52.5)	42±2.78	
Stage 3	28 (12.8)	13 (46.4)	19±4.86	
ASCT performed				0.028
Yes	95 (43.6)	73 (76.8)	45±5.54	
No	123 (56.4)	45 (36.6)	31±4.51	
HRR (cutoff: 0.61)				0.050
Low (≤0.61)	100 (45.9)	57 (57)	32±2.99	
High (>0.61)	118 (54.1)	61 (51.7)	45±4.64	
Beta-2 microglobulin (mg/L)				0.495
≤5.5	114 (52.3)	73 (64)	40±2.64	
>5.5	104 (47.7)	45 (43.3)	33±8.5	

Significant differences in OS were observed across ISS stages (p=0.002). Subgroup analysis showed that patients in ISS stages 1 and 2 had significantly longer OS compared to those in stage 3. A similar trend was observed with R-ISS stages, where stage 3 patients had significantly shorter OS (p=0.001). Notably, no significant difference in OS was observed between high and low RDW groups at diagnosis (p=0.412) (Table 4, Figure 1b).

**Table 4:** OS outcomes of patients ^x^n=given as the number and ratio among 218 people ^y^Given as the number and ratio within the number of patients at risk ^e^Log-rank test Low RDW (≤16.5%) represents patients with RDW ≤16.5%, while high RDW (>16.5%) represents patients with RDW >16.5%. OS times are presented as median ± standard error. ISS: international staging system, R-ISS: revised international staging system, ASCT: autologous stem cell transplantation, RDW: red cell distribution width; OS: overall survival; HRR: hemoglobin/RDW ratio

Parameter	Patients at risk, n(%)^x^	Number of deaths, n(%)^y^	OS (months)	p-value^e^
Age (years)				0.003
≤60	96 (44)	49 (51)	87.8±15.17	
>60	122 (56)	74 (60.7)	41.2±8.3	
Diagnosis				0.688
IgG myeloma	119 (54.6)	67 (56.3)	57.8±11.94	
IgA myeloma	43 (19.7)	28 (58.1)	61.5±13.34	
Light chain myeloma	52 (23.9)	27 (51.9)	49.3±21.04	
Others	4 (1.8)	4 (100)	27.1±13.3	
Hemoglobin (g/dL)				0.800
Low (≤10)	105 (48.2)	57 (54.3)	58.5±11.64	
High (>10)	113 (51.8)	66 (58.4)	56.2±10.14	
RDW groups				0.412
Low	135 (61.9)	73 (54.1)	56.2±11.43	
High	83 (38.1)	50 (60.2)	57.8±10.22	
ISS				0.002
Stage 1	36 (16.5)	17 (47.2)	88±14.65	
Stage 2	70 (32.1)	37 (52.9)	74.8±13.68	
Stage 3	112 (51.4)	69 (61.6)	27.7±8	
R-ISS				0.001
Stage 1	28 (12.8)	13 (46.4)	88.8±11.17	
Stage 2	162 (74.3)	89 (54.9)	56.8±9.49	
Stage 3	28 (12.8)	21 (75)	14.1±6.14	
Chronic kidney disease				0.022
Yes	20 (9.2)	14 (70)	18.4±11.94	
No	198 (90.8)	109 (55.1)	58.1±8.54	
ASCT performed				<0.001
Yes	95 (43.6)	41 (43.2)	114.3±15.06	
No	123 (56.4)	85 (66.7)	20.8±5.32	
HRR (cutoff: 0.61)				0.515
Low (≤0.61)	100 (45.9)	58 (58)	52.7±9.86	
High (>0.61)	118 (54.1)	65 (55.1)	56.8±12.2	
Beta-2 microglobulin (mg/L)				<0.001
≤5.5	114 (52.3)	58 (50.9)	76.4±12.12	
>5.5	104 (47.7)	65 (62.5)	25.1±4.54	

The HRR was also assessed as a prognostic marker. Patients in the high HRR group had a median PFS of 45 months compared to 32 months in the low HRR group. However, neither the difference in PFS (p=0.05) nor in OS was statistically significant (Table 3, Table 4).

Changes in RDW levels from diagnosis to first relapse had a significant impact on survival outcomes (Table 5). Patients with persistently low RDW had the longest PFS (37 months) and OS (88.8 months), whereas those transitioning from low to high RDW had the shortest PFS (nine months) and OS (40.6 months) (p<0.001). At relapse, patients with low RDW showed significantly better outcomes compared to those with high RDW (PFS: 34 vs. 14 months; OS: 81.2 vs. 40.6 months, p<0.001) (Figure 1c, Figure 1d). However, no statistically significant differences were observed in RDW groups at relapse or in changes in RDW levels between diagnosis and relapse based on different chemotherapy regimens (p=0.545 and p=0.201, respectively). Conversely, RDW changes measured at diagnosis and pre-ASCT did not significantly affect survival outcomes (PFS: p=0.964; OS: p=0.503) (Table 6).

**Table 5:** Analysis of PFS and OS based on RDW change from diagnosis to first relapse ^x^Given as the number and ratio within the number of relapses ^e^Log-rank test Low RDW (≤16.5%) represents patients with RDW ≤16.5%, while high RDW (>16.5%) represents patients with RDW >16.5%. Both OS and PFS times are presented as median ± standard error or mean ± standard error, as appropriate. RDW: red cell distribution width, PFS: progression-free survival, OS: overall survival

	Relapses, n(%)	PFS (months)	Deaths^x^, n(%)	OS (months)	p-value^e^ (PFS)	p-value^e^ (OS)
RDW change					<0.001	0.008
High→high	19 (16.20)	22±7.98	13 (68.40)	49.3±13.48		
High→low	25 (21.40)	31±3.72	13 (52)	81.2±16.47		
Low→high	16 (13.70)	9±2.00	11 (68.80)	40.6±13.65		
Low→low	57 (48.70)	37±4.85	28 (49.10)	88.8±16.38		
First relapse RDW					<0.001	<0.001
Low	82 (70.10)	34±3.70	41 (50)	81.2±13.79		
High	35 (29.90)	14±2.96	24 (68.60)	40.6±9.21		

**Table 6:** Analysis of PFS and OS based on RDW change from diagnosis to pre-ASCT ^x^n=given as the number and ratio among 94 people ^y^Given as the number and ratio within the number of patients at risk ^e^ Log-rank test Low RDW (≤16.5%) represents patients with RDW ≤16.5%, while high RDW (>16.5%) represents patients with RDW >16.5%. Both OS and PFS times are presented as median ± standard error or mean ± standard error, as appropriate. RDW: red cell distribution width, ASCT: autologous stem cell transplantation, PFS: progression-free survival, OS: overall survival

	Patients at risk^x^, n(%)	Relapses, n(%)	PFS (months)	Deaths^y^, n(%)	OS (months)	p-value^e^ (PFS)	p-value^e^ (OS)
RDW change						0.964	0.503
High→high	12 (12.80)	9 (75)	38±19.05	5 (41.70)	93.23±10.32		
High→low	15 (16)	12 (80)	38±9.02	9 (60)	79.69±9.15		
Low→high	13 (13.80)	10 (76.90)	54±7.30	3 (23.10)	112.99±14.44		
Low→low	54 (57.40)	42 (77.80)	42±6.70	24 (44.40)	94.83±6.69		
Pre-ASCT RDW						0.612	0.215
Low	69 (73.40)	54 (78.30)	41±4.92	33 (47.80)	79.29±5.60		
High	25 (26.60)	19 (76)	53±7.03	8 (32)	99.27±10.20		

These findings highlight the prognostic importance of dynamic changes in RDW, particularly around relapse, rather than baseline RDW levels alone. Elevated RDW at diagnosis was associated with more advanced disease stages, as evidenced by R-ISS, but not with independent survival outcomes. Additionally, ASCT and higher hemoglobin levels were critical factors for improving PFS and OS in MM patients.

## Discussion

Our retrospective analysis of 218 MM patients demonstrates a strong association between elevated RDW levels at diagnosis and advanced disease stages, particularly R-ISS stage 3. Furthermore, dynamic changes in RDW from diagnosis to first relapse were closely linked to survival outcomes, underscoring RDW as a pivotal biomarker for both disease severity and prognosis, especially in the context of relapse.

Several studies have explored the prognostic role of RDW in MM. Lee et al. analyzed 146 symptomatic myeloma patients and categorized RDW levels into high (>14.5%) and normal (≤14.5%) groups, finding that patients with ISS stage 1 disease had significantly lower RDW compared to those in ISS stages 2 and 3 (p<0.001) [13]. Similarly, Wang et al. studied 196 MM patients, setting the upper RDW limit at 18.05%, and observed that high RDW levels were associated with low platelet counts, low hemoglobin, and elevated lactate dehydrogenase (LDH) levels [14]. Meng et al., in their analysis of 166 MM patients, used a cutoff of 14% and reported that high RDW correlated with poor prognostic factors such as low hemoglobin, low platelet counts, increased sedimentation rate, low albumin, elevated LDH, high creatinine and calcium levels, and increased bone marrow plasma cell infiltration (p<0.05) [15]. Zhou et al. further demonstrated that high RDW levels were associated with low hemoglobin, low albumin, fatigue, bone marrow plasma cell infiltration, and advanced ISS stage [16].

Another retrospective study involving 190 newly diagnosed MM patients found that RDW levels were significantly higher in IgA MM and progressively increased from ISS stage 1 to 3. RDW was also associated with lower albumin values, higher beta-2 microglobulin and LDH levels, and more bone marrow plasma cell infiltrates, reinforcing its role as a marker of disease burden and aggressiveness [17]. Our findings align with these studies, as R-ISS stage 3 and IgA myeloma were significantly more frequent in the high RDW group at diagnosis (p=0.022 and p=0.040, respectively). However, the lack of standardized RDW cutoff values across laboratories complicates the establishment of a universal prognostic threshold, underscoring the need for future large-scale studies with uniform methodologies.

In terms of survival outcomes, studies by Lee et al. [13] and Zhou et al. [16] reported no significant differences in OS between high and low RDW groups. However, in both studies, patients in the low RDW group had longer PFS (p=0.029, p=0.021, respectively). Similarly, Meng et al. observed significant differences in both PFS and OS, with median PFS of 27 and 36 months (p=0.038) and OS of 45 and 58 months (p=0.021) in the high and low RDW groups, respectively [15]. Wang et al. reported better OS in patients with low RDW at diagnosis (50 months vs. 40 months, p=0.010) [14]. A recent meta-analysis of 1165 patients from nine studies further confirmed that elevated RDW is significantly associated with poor prognosis in MM (OS: HR=1.91; PFS: HR=2.87) [18]. However, unlike previous studies, we did not observe significant differences in PFS or OS between the low and high RDW groups at diagnosis (p=0.561 and p=0.412, respectively) [13,15,16]. This discrepancy could stem from differences in RDW cutoff values, variations in chemotherapy regimens, or the retrospective nature of our data collection.

ASCT significantly improved survival outcomes in our study, reducing the risk of death by 51%. Patients with lower RDW levels at diagnosis were more likely to undergo ASCT (p=0.010). While ASCT significantly improved OS but not PFS in some studies [13], other analyses demonstrated its positive impact on both PFS and OS in MM [19]. Lee et al. similarly found that ASCT was an independent factor for OS (p<0.001) [13]. Given that RDW tends to increase with advanced disease stages and poor prognostic factors, our findings support its consideration as an additional marker in selecting ASCT candidates [17,18].

The HRR has also been proposed as a prognostic marker in MM. Baysal et al. demonstrated that higher HRR values were associated with significantly longer survival (p=0.01) and were an independent risk factor (p=0.002) [12]. In our study, HRR showed a similar trend, with longer PFS in the high HRR group (45 months vs. 32 months, p=0.05), though no significant difference in OS was observed. Several studies suggest HRR as a prognostic marker for malignancies, further supporting its potential value in MM [20,21].

A major limitation of this study is its retrospective design. Additionally, inconsistencies in RDW cutoff values across studies hinder direct comparisons and the development of standardized prognostic thresholds. Future research should prioritize prospective, multi-center trials with uniform RDW measurements and integrate RDW into predictive models alongside established biomarkers to improve risk stratification and enable more personalized treatment approaches for MM patients.

## Conclusions

Our study highlights the prognostic significance of RDW in MM, emphasizing its dual role at diagnosis and during first relapse. Although no significant differences in PFS or OS were observed between RDW groups at diagnosis, lower RDW levels were associated with early-stage disease and a higher likelihood of undergoing ASCT. Importantly, dynamic changes in RDW from diagnosis to first relapse demonstrated a strong prognostic impact, with reductions in RDW correlating with significantly improved PFS and OS outcomes.

The variability in RDW thresholds across studies and the multifactorial factors influencing RDW levels remain key challenges in standardizing its prognostic value. These findings underscore the necessity for future research to establish uniform RDW cutoffs and to integrate RDW dynamics into comprehensive prognostic models alongside established clinical and molecular markers. Such advancements could enhance risk stratification, enable more personalized treatment planning, and ultimately improve outcomes for MM patients.
